# Outbreak of Natural Severe Fever with Thrombocytopenia Syndrome Virus Infection in Farmed Minks, China

**DOI:** 10.3201/eid3006.240283

**Published:** 2024-06

**Authors:** Ying Wang, Mingfa Yang, Hong Zhou, Chuansong Quan, Hongtao Kang

**Affiliations:** Harbin Veterinary Research Institute, Chinese Academy of Agricultural Sciences, Harbin, China (Y. Wang, M. Yang, H. Kang);; Shandong First Medical University and Shandong Academy of Medical Sciences, Taian, China (H. Zhou, C. Quan)

**Keywords:** viruses, vector-borne infections, severe fever with thrombocytopenia syndrome virus, SFTSV, farmed minks, fatal symptoms, public health problem, tickborne virus, vector-borne infections, ticks, China

## Abstract

We isolated severe fever with thrombocytopenia syndrome virus (SFTSV) from farmed minks in China, providing evidence of natural SFTSV infection in farmed minks. Our findings support the potential role of farmed minks in maintaining SFTSV and are helpful for the development of public health interventions to reduce human infection.

Severe fever with thrombocytopenia syndrome (SFTS) is an emerging disease caused by a novel tickborne bunyavirus, SFTS virus (SFTSV), which was first identified in China in 2009 (*1*). Outside of China, SFTS was subsequently reported in South Korea, Japan, Vietnam, Myanmar, and Pakistan and now poses a global health problem (*2*–*6*). SFTSV is an enveloped virus belonging to the genus *Bandavirus*, family *Phenuiviridae*, order *Bunyavirales*. The virus has 3 single-stranded negative-sense RNA segments, large (L), medium (M), and small (S) (*1*). The *Haemaphysalis longicornis* tick is widely considered to be the primary transmission vector (*7*), but the natural animal hosts of SFTSV remain uncertain. Despite the high seroprevalence observed in various domestic animals in SFTSV-endemic regions, such as goats, cattle, dogs, pigs, chickens, and rodents, many of those animals do not show notable symptoms. Those infections were plausibly a result of SFTSV transmission from infected ticks (*8*).

We describe an outbreak of SFTS on a mink farm situated in Shandong, China. During late May through early July, 2022, >1,500 minks on this farm exhibited symptoms such as vomiting, diarrhea, and, in a small number, limb convulsions. Most minks exhibiting clinical manifestations died of the disease (Figure 1, panel A). Clinical manifestations included loose stools, mesenteric lymph node enlargement, and hyperemia (Figure 1, panels B and C), consistent with typical enteritis symptoms. Treatment with multiple antibiotics was not effective, and mink enteritis virus infection was ruled out through testing with a colloidal gold immunochromatographic assay. Intestinal tissue samples from minks with diarrhea (n = 10) were collected and forwarded to the Harbin Veterinary Research Institute, Chinese Academy of Agricultural Sciences, Harbin, China, for testing.

**Figure 1:**
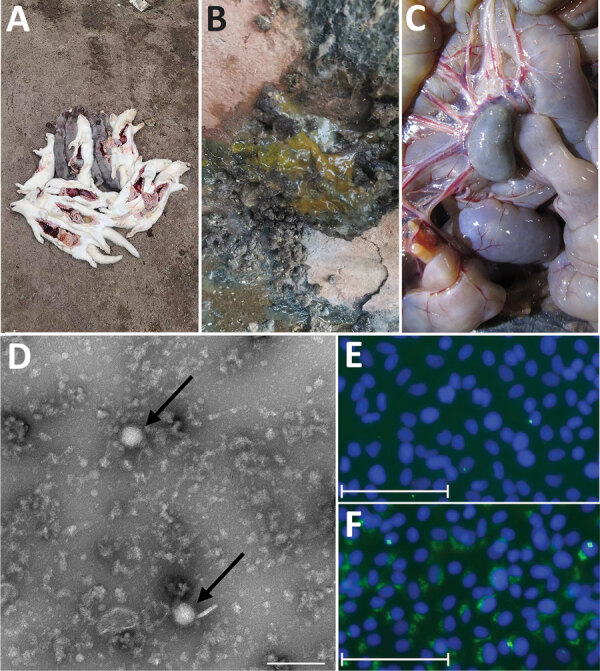
Clinical manifestations, TEM analysis, and immunofluorescence assay findings in outbreak of natural severe fever with thrombocytopenia syndrome virus (SFTSV) infection in farmed minks, China. A–C) Clinical manifestations were death (A), loose stools (B), and enlargement and hyperemia of the mesenteric lymph nodes (C). D) Virus detection by TEM analysis showed typical enveloped virions (arrows). Scale bar indicates 200 nm. E, F) Immunofluorescent pictures of Vero cells infected with SFTSV SD01/China/2022 isolate. Differences between the blank control (E) and green fluorescence (F) indicates SFTSV particles in the monolayer of Vero cells. Scale bars in panels E and F indicate 75 μm.

To identify the causative pathogen, we tested tissue samples for mink enteritis virus, Aleutian mink disease virus, carnivore rotavirus, carnivore coronavirus, and SFTSV. Among those, only tests for SFTSV were positive; cycle threshold (Ct) values were 18–22 (positive samples Ct <36) using SFTSV-specific primers (*9*). We obtained the SFTSV isolate from the intestinal tissue samples through a series of blind passages. We visualized virus particles by negative staining and transmission electron microscopy analysis following a discontinuous sucrose gradient purification. Virus particles presented the typical morphology of bunyavirus, as enveloped spherical particles with an average diameter of 100 nm (Figure 1, panel D). We then used immunofluorescence assays to analyze SFTSV nucleoprotein expression in Vero cells. We observed the green fluorescence by using an inverted fluorescence microscope. The results showed that SFTSV nucleoprotein expression was detected in Vero cells (Figure 1, panels E and F). Those results together confirmed the isolate, named SD01/China/2022, was SFTSV.

To obtain the complete genome of the SFTSV SD01/China/2022 isolate, we performed metagenomic next-generation sequencing to the sequence the RNA of the full-length genome. In brief, we constructed RNA sequencing libraries using the MGIEasy mRNA Library Prep Kit (BGI, https://www.bgi.com). Subsequently, we generated paired-end reads (100 bp) of the RNA libraries on the BGISEQ-500RS sequencing platform (BGI). We determined the complete genomes of SD01/China/2022, submitted to GenBank (accession nos. PP066883–5), and compared them with previously reported SFTSV strains. Phylogenetic analysis suggested that the L, M, and S gene segments of SD01/China/2022 all descended from C3; the L and M gene segments were genetically closest to the SFTSV strains HB2016-003 *Homo sapiens*/Hubei/China/2016 and the S gene segment was closest to the SFTSV strains SDLZDog01/2011 dog/China/2011 and SDPLP01/2011 *Homo sapiens*/China/2011 (Figure 2, panels A–C).

**Figure 2:**
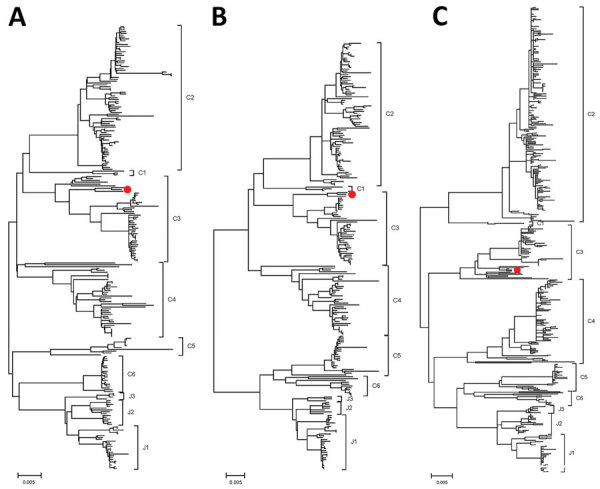
Phylogenetic analysis of natural severe fever with thrombocytopenia syndrome virus SD01/China/2022 isolate from outbreak in farmed mink, China. Phylogenetic trees were based on the alignment of large (A), medium (B), and small (C) gene segment sequences from the isolate from this study (red circles). The maximum-likelihood method based on the Tamura-Nei model was used to analyze the molecular evolution by using MEGAX (https://www.megasoftware.net). The conﬁdence of the resulting trees was evaluated by 1,000 bootstrap replications. All other parameters were used as default. Scale bars indicate nucleotide substitutions per site.

Previous research has shown the presence of antibodies to the nucleoprotein of SFTSV in farmed minks and suggested that minks were infected with SFTSV in China (*10*). In this study, we successfully isolated and identified an SFTSV isolate, named SD01/China/2022, in farmed minks in China. The symptoms of farmed minks in this case were consistent with SFTS symptoms, such as gastrointestinal disorders and central nervous system manifestations, which proved the occurrence of natural SFTSV infection–related fatalities in this population. Our findings reveal the threat of SFTS to the fur animal–breeding industry. Of note, phylogenetic analysis of the isolate indicated high homology with SFTSV strains in humans, suggesting that the viruses generally infected both humans and minks, further supporting the potential role of farm minks in maintaining SFTSV. Farmed minks have potential for direct contact with humans and might serve as crucial amplifying hosts in the transmission of SFTSV. Further analysis of SFTSV infection in other captive fur animals, such as raccoon dogs and foxes, will be required to determine other key reservoirs for SFTSV. We recommend a focus on the registration of mink exposure for humans with SFTS-like illnesses, as well as increased measures to reduce SFTSV exposure risk. 

Appendix Additional information about outbreak of natural severe fever with thrombocytopenia syndrome virus infection in farmed minks, China
