# Supplementary material for: Outbreak of Natural Severe Fever with Thrombocytopenia Syndrome Virus Infection in Farmed Minks, China
Source: Emerg Infect Dis. 2024 Jun;30(6):1299–301. doi: 10.3201/eid3006.240283 (PMC11139004; doi:10.3201/eid3006.240283)
Supplement: Appendix — Additional information about outbreak of natural severe fever with thrombocytopenia syndrome virus infection in farmed minks, China [file 24-0283-Techapp-s1.pdf]

Article DOI: https://doi.org/10.3201/eid3006.240283

EID cannot ensure accessibility for Supplemental Materials supplied by authors. Readers who have difficulty accessing supplementary content should contact the authors for assistance.

Outbreak of Natural Severe Fever with Thrombocytopenia Syndrome Virus Infection in Farmed Minks, China

Appendix.

A

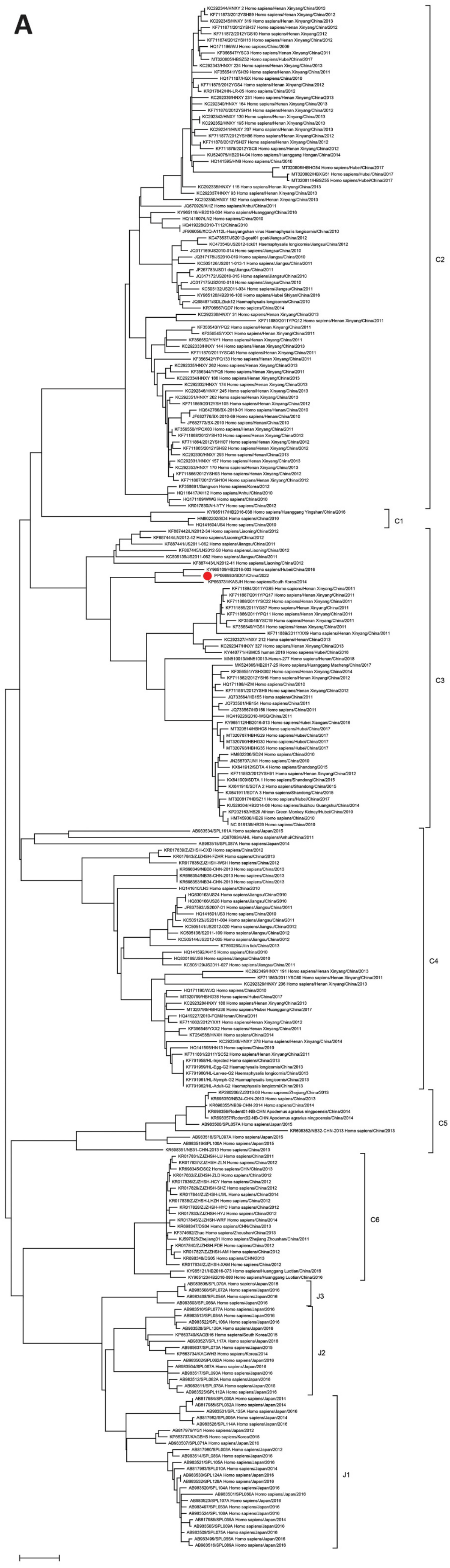

B

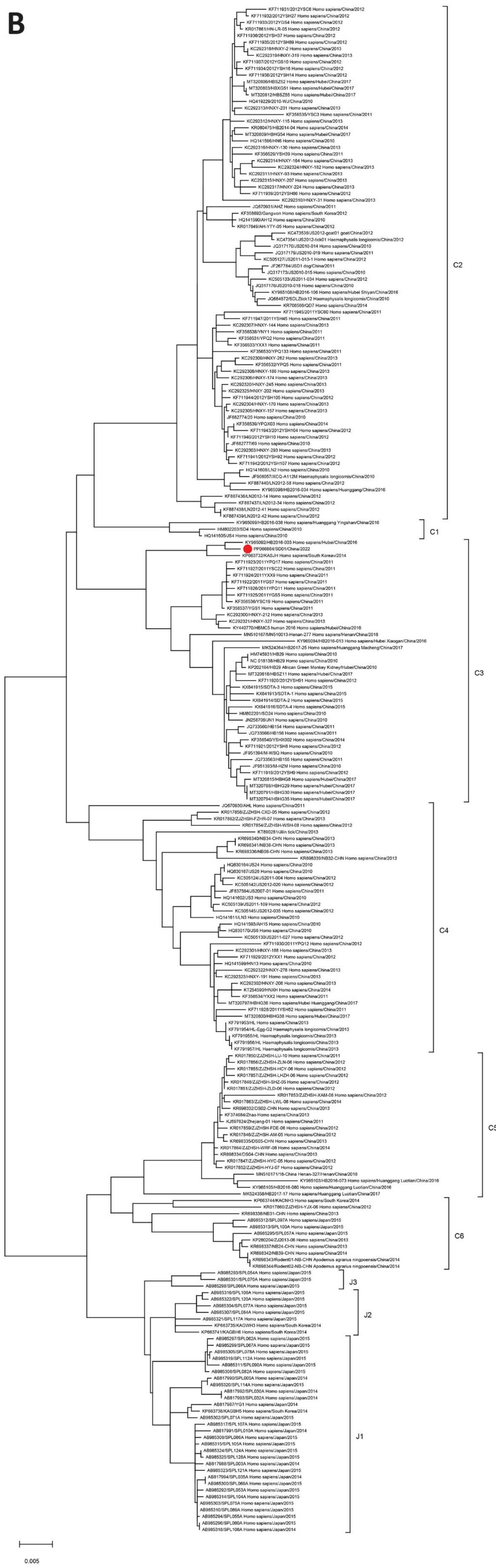

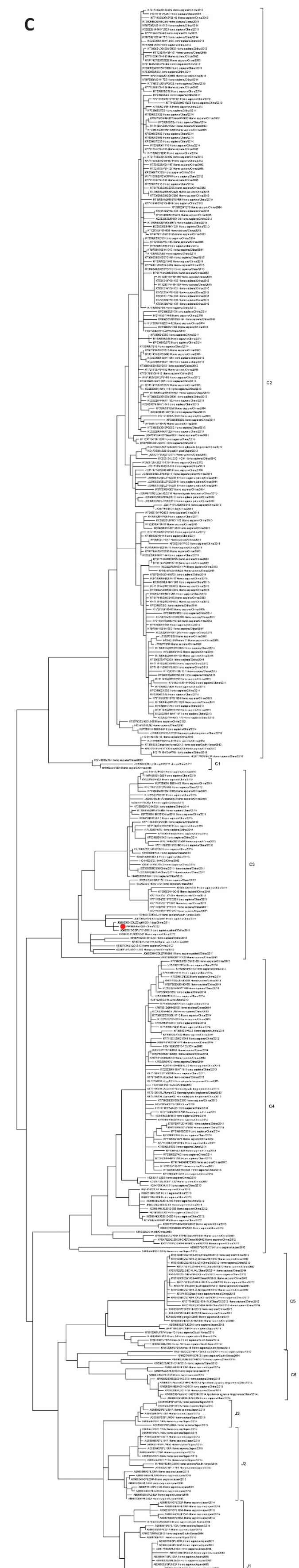
C
C2
C1
C3
C4
C5
C6
J3
J2
J1

Appendix Figure. Phylogenetic analysis of SFTSV sequences of the L (A), M (B) and S (C) gene segments with name of the isolate, location and accession number.
